# Stop the Divide: Facilitators and Barriers to Uptake of Digital Health Interventions Among Socially Disadvantaged Populations

**DOI:** 10.31486/toj.22.0101

**Published:** 2023

**Authors:** Eboni G. Price-Haywood, Connie Arnold, Jewel Harden-Barrios, Terry Davis

**Affiliations:** ^1^ Ochsner-Xavier Institute for Health Equity and Research, New Orleans, LA; ^2^ Department of Research, Ochsner Clinic Foundation, New Orleans, LA; ^3^ The University of Queensland Medical School, Ochsner Clinical School, New Orleans, LA; ^4^ Department of Medicine, Louisiana State University Health Sciences Center–Shreveport, Shreveport, LA

**Keywords:** *Digital divide*, *digital technology*, *patient portals*, *remote consultation*, *socioeconomic disparities in health*, *telemedicine*

## Abstract

**Background:** The coronavirus disease 2019 pandemic ushered in rapid adoption of telehealth services. This study examines patient and provider experience and provides recommendations for facilitating the use of digital health interventions among socially disadvantaged populations.

**Methods:** This qualitative study was conducted from May to July 2021 via semistructured interviews. Forty patients and 30 primary care providers (PCPs) in Louisiana were recruited within an integrated delivery health system and a rural health center. Technology acceptance models were used to develop a thematic coding scheme.

**Results:** Most patients self-identified as Black (67.5%) and female (72.5%), had a mean age of 51 years, lived in an urban area (76.9%), and had Medicaid (57.9%). Most PCPs were White (79.3%) and male (51.7%), had a mean age of 39 years, and reported Medicaid as the predominant insurer (58.6%). Patient use of smartphones for internet access to health and nonhealth activities was common. PCPs noted the need to address misinformation or misinterpretation of information on the internet. Most patients had used a patient portal (72.5%) and noted the convenience of messaging. PCPs reported large increases in messaging workloads. Most patients had had telemedicine visits (65.6%); however, Wi-Fi/broadband problems limited video visits. PCPs expressed concerns regarding the types of chief complaints that are appropriate for telemedicine visits and reported workflow inefficiencies when clinic sessions had mixed visit types. Patients and PCPs valued remote telemonitoring as adjuncts to care; however, limited service availability and insurance coverage were barriers.

**Conclusion:** Infrastructure barriers (broadband, insurance) and workload imbalance temper enthusiasm for using digital health solutions. Health systems should implement complementary patient and provider user-centric strategies for facilitating uptake of technology.

## INTRODUCTION

The coronavirus disease 2019 (COVID-19) pandemic ushered in rapid adoption of telehealth services in the United States^1^ and shed light on the ability of health systems to quickly adjust health care delivery models during crises. Telehealth, defined as the use of electronic information and telecommunications technologies to support long-distance clinical care,^2^ has 2 forms: (1) 2-way synchronous interactive communication via audio and visual equipment (eg, telemedicine) and (2) asynchronous interactions via various technologies (eg, patient portals, email/text messaging, mobile applications [apps], sensors/tracking devices). Notably, disparities in access to and use of telehealth among medically underserved, socially disadvantaged populations were observed during the pandemic. In a 2021 national survey of households in the United States, 1 in 4 respondents reported having used telehealth services in the prior 4 weeks.^3^ The highest rates of telehealth usage were among individuals who had Medicaid/Medicare insurance, those with an annual income <$25,000, and those who identified as Black. Rates were lowest among individuals who were uninsured and young adults ages 18 to 24 years. Significant disparities were seen in audio vs video telehealth use. Video telehealth rates were highest among young adults, White individuals, and those with private insurance and/or incomes of at least $100,000. The lowest rates of video telehealth were among individuals who reported less than a high school education, those who were 65 years and older, and racial/ethnic minorities.

Regarding asynchronous telecommunication, the 2020 Health Information National Trends Survey (HINTS) of US adults demonstrated that rates at which individuals were offered access to their online medical record via a patient portal and subsequently accessed their record increased between 2014 and 2020 by 17% and 13%, respectively; however, no significant increases occurred between 2019 and 2020.^4^ Individuals whose providers encouraged them to use patient portals used their online medical record at higher rates than those who were not encouraged. Approximately 4 in 10 portal users accessed their records through a smartphone app. Individuals who accessed their portals via smartphone and computer had higher rates of portal use compared to those who accessed their portals via only 1 method. A 2021 Pew Research Center study demonstrated that while Black and Hispanic adults were less likely to own a traditional computer or have high-speed internet at home compared to White adults, there were no differences in access to smartphones and tablets.^5^ Rural adults, however, were less likely to have home broadband or to own a mobile device or traditional computer compared to urban and suburban adults.^6^

Expansion of remote physiologic monitoring (RPM) services was made easier with updates to the Centers for Medicare and Medicaid Services policy for reimbursement.^7^ RPM facilitates management of acute and chronic conditions by transmission of electronically collected data (eg, blood pressure, blood glucose, weight) that are automatically uploaded via an approved device to a secure location (eg, electronic medical record) where the data are available for analysis and interpretation by a clinical provider. Nonetheless, the uptake of RPM was limited. In the 2020 HINTS study, few portal users transmitted health data to a service or app.^4^ Kirkland et al suggest that socioeconomic status and clinic location impact the level of engagement with data transmission.^8^ Fritz et al further suggest that race and neighborhood disadvantage impact patients’ choice of RPM program type (telephone-based vs patient portal app).^9^

Notwithstanding the numerous barriers to equitable access to virtual care options, telehealth is here to stay. Therefore, health systems should find effective ways to mitigate the digital divide. We conducted this study to gain insights from primary care clinicians and their patients who are users or nonusers of telehealth services. The main objectives of this study were to (1) compare patient and primary care provider (PCP) perceptions of facilitators and barriers to engagement in using patient portals, remote telemonitoring, and/or telemedicine in medically underserved communities; and (2) recommend strategies for effectively facilitating interactive patient-provider use of these interventions among socioeconomically disadvantaged and medically underserved populations.

## METHODS

### Study Setting, Population, and Design

This qualitative study was conducted at Ochsner Health, Louisiana's largest nonprofit, academic, multispecialty health care delivery system, between May and July 2021 (during the COVID-19 pandemic). The study investigators targeted 8 clinics located in north and southeast Louisiana that serve a large Black/African American and/or Medicaid-/Medicare-insured population. These clinics included 5 in Shreveport and Monroe and 3 in the New Orleans metropolitan area. All clinics were located in federally designated medically underserved areas as defined by the US Department of Health and Human Services. Two clinics are part of a rural federally qualified health center in North Louisiana; all the others are primary care practices owned or managed by Ochsner Health. The study recruited PCPs (physicians and nurse practitioners) who work at the targeted clinics in internal medicine, family medicine, or medicine-pediatric specialties and who had either self-referred themselves to the study or were recommended by clinic management as key stakeholders. PCPs recruited in the New Orleans area were early adopters of incorporating telehealth into their clinical practices. In contrast, PCPs in Shreveport and Monroe were mostly new users at the time of this study. Patients receiving care at the target clinics were recruited through a variety of mechanisms to obtain a convenience sample for the study: (1) MyChart recruitment notices, (2) onsite recruitment, (3) PCP referrals, and (4) referrals from study participants. This study was approved by the Ochsner Health Institutional Review Board (IRB), with IRB acknowledgement from the Louisiana State University Health Sciences Center–Shreveport.

### Digital Health Technology

In 2012, Ochsner Health implemented the Epic electronic medical record (Epic Systems Corporation) that includes the MyChart patient portal. To date, approximately 925,564 patients have activated their patient portal accounts within the entire health system across Louisiana, Mississippi, Alabama, and the Gulf South. The portal can be accessed via mobile device (eg, smartphone, tablet) or computer with internet service. In 2015, Ochsner launched digital medicine programming in the New Orleans area for outpatient chronic care management of hypertension, diabetes, hyperlipidemia, and chronic obstructive pulmonary disorder, as well as antepartum and postpartum care.^10^ The digital medicine program includes health coaches for lifestyle counseling and clinical pharmacists for medication management under a collaborative drug therapeutic management agreement with PCPs. These programs were launched in Ochsner clinics in North Louisiana in 2020. Participation in these programs requires activation of patient portal accounts and remote monitoring equipment capable of syncing with MyChart (blood pressure cuff, glucometer, weight scale, spirometer). More recently, Ochsner launched telemedicine virtual visits with a rapid escalation of use during the COVID-19 pandemic in 2020. Virtual visits are also conducted via audiovisual connection through MyChart.

The rural health center clinics use multiple platforms for telehealth. For patient portal access, the clinics use the AthenaNet portal system that is tethered to the AthenaHealth cloud-based electronic medical record system.^11^ For telemedicine, the clinics used StarLeaf, a messaging, meeting, and calling platform.^12^ Prior to the COVID-19 pandemic, the rural health center clinics piloted the Esvyda platform (ESVYDA! Inc.) for RPM of hypertension and diabetes.^13^ At the time of this report, a registered nurse had been hired to formally launch the RPM program.

### Participant Survey

Study participants completed online surveys or structured telephone interviews to self-report demographic data prior to the conduct of interviews or focus groups. Self-reported age, sex, and race were collected from patients and PCPs. For patients, additional survey items included urban/rural status, primary insurance, use of the patient portal within the last 12 months, and use of telemedicine (video and/or audio only) during the COVID-19 pandemic. For PCPs, survey items also included type of provider (physician or nurse practitioner), level of training for physicians (resident, teaching faculty/staff), and estimated percentages for the most common insurance type and for use of the patient portal among the PCPs’ assigned patient panels.

### Semistructured Interviews and Focus Groups

All interviews and focus groups were conducted by 2 health services research faculty experienced in qualitative methods. The investigators developed a moderator's guide that was used to structure the discussion. Open-ended questions were asked to assess experience, attitudes, satisfaction, and challenges with using health technology. Twenty-eight interviews were conducted via telephone. Six focus groups were conducted in person or via Zoom (4 provider groups with 27 participants; 2 patient groups with 15 participants). All interviews and focus groups were conducted in English, audio-taped, and transcribed verbatim. In addition, an investigator took notes of all sessions, and the notes were later organized by theme. The discussions explored perceptions of patient use of smartphones, mobile devices, and computers; patient use of mobile device apps and internet for health and non-health-related activities; provider/patient use of the patient portal, digital medicine programs, and/or telemedicine; and the benefits and challenges of using these technologies from their perspectives. For patients who were nonusers of a given technology, the discussions explored reasons for nonusage and perceptions of potential facilitators or barriers to use.

### Qualitative Data Analysis

The team used the technology acceptance model and the unified theory of acceptance and use of technology model as frameworks for developing a thematic coding scheme.^14^ Key concept domains within these models include behavioral intention (defined as motivation or willingness to use technology); attitude (defined as evaluative judgment of technology use); perceived ease of use (defined as perception of minimal effort to use); perceived usefulness (defined as perception that technology will enhance experience); social influence (defined as important social contacts, such as family, believe technology should be used); and perceived behavioral control (subdomains include self-efficacy, facilitating conditions, and controllability). Facilitating conditions are factors that facilitate or impede technology use such as skills, resources, and technical support. Controllability reflects perceptions of the amount of control one has to use technology. Emerging themes that did not clearly fit into the model concepts were broadly coded as benefits/facilitators or challenges/barriers to technology use. One member of the research team (EPH) served as the primary coder, and 2 investigators served as secondary reviewers (CA, TD). The secondary reviewers used their session notes to guide their review of the primary coding scheme. Upon consensus of coded themes, perspectives of patients were compared to those of the PCPs for similarities and differences in common themes. NVivo 12 software (QSR International) was used to organize, store, analyze, and visualize data.

## RESULTS

### Participant Characteristics

A total of 40 patients and 30 providers participated in the study (Table 1). Most patients were middle-aged, female, Black/African Americans who live in urban areas, were insured by Medicaid, and had used a patient portal and/or telemedicine services. Most PCPs were younger White male physicians who reported serving mostly publicly insured populations (Medicaid/Medicare). Among all interviews (n=28) and focus group discussions (n=6), the frequencies of technology acceptance themes were 51.2% attitude, 44.8% facilitating conditions, 34.5% perceived ease of use, 27.6% behavioral intention, 24.1% perceived usefulness, 20.7% self-efficacy, 6.9% controllability, and 6.9% social influence.

**Table 1.** Study Participant Self-Reported Characteristics

Study Group/Characteristic	Value
**Patients, n=40**	
Age, years, mean ± SD, n=37	51 ± 13.2
Female	29 (72.5)
Race	
** **White	12 (30.0)
** **Black or African American	27 (67.5)
** **Other	1 (2.5)
Lives in the city, n=39	30 (76.9)
Receives care at a rural federally qualified health center	4 (10.0)
Insurance, n=38	
** **Medicaid	22 (57.9)
** **Medicare	11 (29.0)
** **Commercial	4 (10.5)
** **None	1 (2.6)
Used the patient portal within the past 12 months	29 (72.5)
Used telemedicine services during the pandemic, n=32	21 (65.6)
**Primary care providers, n=29** ^a^	
Age, years, mean ± SD	39 ± 11.3
Female	14 (48.3)
Race	
** **White	23 (79.3)
** **Black or African American	3 (10.3)
** **Other	3 (10.3)
Type of primary care provider	
** **Physician^b^	26 (89.7)
** **Nurse practitioner	3 (10.3)
Works in a rural federally qualified health center	4 (13.8)
Predominant insurance type served by clinic	
** **Medicaid	17 (58.6)
** **Medicare	8 (27.6)
** **Commercial	2 (6.9)
** **Other	2 (6.9)
Estimated percentage of patients using the patient portal	
** **<25%	15 (51.7)
** **25% to 49%	10 (34.5)
** **≥50%	4 (13.8)

^a^Demographic data are missing for 1 provider.

^b^Includes 14 resident physicians.

Note: Data are presented as n (%) unless otherwise indicated.

### General Use of Technology

Approximately 60% of interview and focus group discussions provided insights into patients’ use of the internet to search for health information such as diagnosing symptoms or looking up medication side effects. The Google search engine was the most frequently cited source of information (Figure). Common challenges that the PCPs voiced included the downstream consequence of misinformation or misinterpretation of information on the internet and the need to help educate patients about which online resources are reliable. One-third of the interview/focus group discussions revealed patients’ common use of the internet for banking, shopping, paying bills, and other activities and preferential use of their smartphones to access the internet, email, and various apps. While smartphone access was very common, knowledge of how to use various features of these mobile devices varied. Additionally, where patients lived (rural vs urban) impacted access to high-speed broadband and therefore to the use of digital technology.

**Figure.**
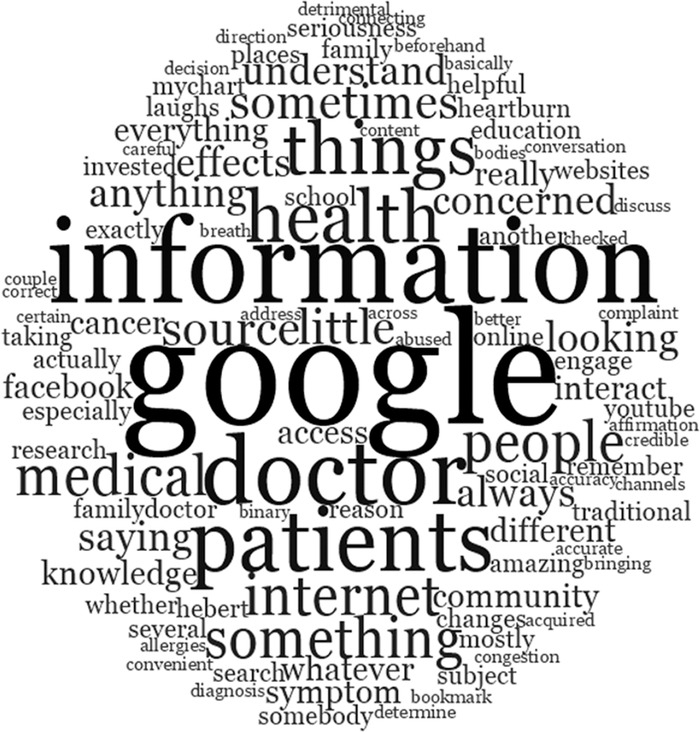
Word cloud of common themes for patient use of the internet for heath information.

### Patient Portal Usage

Table 2 displays similarities and differences in patient and PCP perceptions of electronic medical record–tethered portals. The benefits of using a patient portal were highlighted in 55% of all interviews and focus group discussions. The most notable benefits identified were the consolidation of medical record information that can be easily accessed via mobile devices; the ease of scheduling appointments and requesting medication refills; and the ability to send messages. Descriptions of portal challenges (raised in 37.9% of interviews/focus groups) mostly focused on problems with logging in, remembering passwords, and navigating website/app upgrades. The advantages and disadvantages of the portal messaging tool were highlighted in almost 50% of the interviews/focus groups. Notably, patients appreciated the convenience of medical advice messaging, whereas PCPs lamented the overall large number of messages irrespective of the source of messaging (phone or portal) and care team workload inefficiencies in managing these messages.

**Table 2.** Patient and Provider Perceptions of Patient Portal Usage

Thematic Domain	Patient^a^	Primary Care Provider
Attitude	*User:* When the MyChart thing came, that was just great. I love it. *Nonuser:* Listen, I know how to read, so I’d rather get everything by mail.	I’ve had more elder, more elderly tell me…This whole smartphone thing isn’t for me. I still got a flip phone. But even then, sometimes, they’ll be like I have a daughter that has a computer. I’m going to get her to sign up for it.
Behavioral intention	*User:* I did my routine. I called the nurse and I find out just, you know, like that. So, I don’t know if they’re going to text me or call me with information. They probably would but I never ask.	And I have really found that you can’t judge it by age. I’ve had plenty people in their 20s say they don’t want to be on the portal. I have plenty of patients in their 80s who are on the portal who love it. So, you really can’t judge by age.
Perceived behavioral control (subdomains facilitating conditions and controllability)	*User:* I use MyOchsner mostly on my cellphone because of convenience. (*facilitating conditions)* *Nonuser:* Being home by myself, it's really rough for me with computers and cell phones. And with the COVID, everything is online, online, online, and it frustrates me. *(controllability)*	I look at their chart to say, “Hey, I see you’re active. Are you still able to get on?” Because I don’t want to send them results or messages and then they can’t ever see it. And some of them will say, “Oh, I forgot my username, forgot my password.” Not all of them have, you know, the newest phones where you can use finger touch or face recognition. So the ones that still need to remember that, yes, I find that some of them do have trouble remembering. (*facilitating conditions)*
Perceived ease of use	*User:* It saves talking on the phone or going to the doctor and bothering somebody. You can just push a few flip flops there and wait and hear “Ding dong” when it's ready for you.	It needs to be a lot more visually simple. There should not be a drop-down menu that takes up the entire computer screen.
Perceived usefulness	*User:* So the fact that they had a system that could consolidate all of that…It made it easier for me to manage all my co-pays and all that kind of stuff…I mean, I get my alerts when I have an appointment, my reminders. *User:* The best thing about it, like I said, is keeping in touch with my physicians….my doctor or his nurse is pretty quick on answering these questions. It benefits me a lot, yes.	Some have used a portal in another system and it was pretty worthless. So, when I try to tell them — no, I’m telling you this is a very different much more advanced thing. I think the messaging volume is a lot no matter how they contact us. So, I think there's just a big burden of messages period. But doesn’t matter whether it comes in that way or phone and then I prefer portal versus phone.

^a^*User* indicates that the patient uses digital technology, defined as patient portal, chronic disease remote monitoring, and/or telemedicine.

COVID, coronavirus disease 2019.

### Remote Monitoring of Chronic Diseases

Table 3 displays similarities and differences in patient and PCP perceptions of remote monitoring of chronic diseases. Most patients in the study were not using a remote monitoring program. In contrast, most of the PCPs in the study reported having some patients enrolled in a remote monitoring program. Nonetheless, patients and PCPs appreciated the value of such programming for chronic disease management (thematic domain of perceived usefulness). For patients, self-efficacy and perceived ease of use were prominent themes. PCPs additionally conveyed the importance of patient access to mobile devices and internet/Wi-Fi services, as well as willingness or interest in having someone review their blood pressure or glucose and adjust medications between clinic visits. PCPs also reported lack of insurance coverage for monitoring devices as a major barrier.

**Table 3.** Patient and Provider Perceptions of Chronic Disease Remote Monitoring

Thematic Domain	Patient^a^	Primary Care Provider
Behavioral intention		If they don’t want that accountability and they’re really not ready to do anything, then they’re just going to say, “I’m not going to do that.” But there's some that are excited about it and so they get more engaged.
Perceived behavioral control (subdomain facilitating conditions)		Half the people I’ve put on the hypertension or digital medicine stuff, at least 25% refused because of lack of access to internet. [The readings] won’t upload to Epic until they get connected to Wi-Fi…I think that's part of it. If they are on a plan with limited amount of data or something like that…those charges still apply. That's not free.
Perceived behavioral control (subdomain self-efficacy)	*Nonuser*: My husband would really need something like that. Every time he goes to the doctor, his pressure is high. But once again, we’re in our 60s, and those computer things….	Some of them get gifted smartphones…but I would say even within that, there's not that decision. Like, I’m not going to log in to take my blood pressure, because I don’t want [to]. It's like, I don’t know how, and I don’t have people around me that value that, and so it's more of, like, I can’t do it.
Perceived ease of use	*User*: It beats taking it and writing it down and putting in a log. And then they’ll add to a sheet on MyChart so they can read it.	The hypertension is way easier. It's just a blood pressure check; it uploads. Once you get through that first technology difficulty, get them the cuff, it seems like they can do it better. The touchpoints are less, even though I have a lot of disenrollment because they’re just like, “I can’t deal with the people calling me.” But the diabetes one is, like, orders of magnitude more….
Perceived usefulness	*User*: I take my blood pressure, pulse, blood sugar, temperature, and O2 sat, and I put them all into the app and it directly links to her along with the MyChart. And then if I have an abnormal blood pressure, they’ll send me a text asking me to retake it after 15 minutes and I retake it and it goes back to them. And I can put notes in about why I think my blood pressure was high or, you know, I hadn’t taken my medicine yet.	I have some that have been loyal users. I think the ones that tend to be a little bit more anxious about it and not really as comfortable with taking their blood pressure at home on their own like the touch points. Some people prefer to have someone checking on them if their blood pressure is up or if their sugar goes up or down.

^a^*User* indicates that the patient uses digital technology, defined as patient portal, chronic disease remote monitoring, and/or telemedicine.

app, application; O2 sat, oxygen saturation.

### Telemedicine Usage

Table 4 displays similarities and differences in patient and PCP perceptions of telemedicine. The benefit of telemedicine was a major theme raised in 51% of the interviews and focus group discussions. Key highlights were the convenience of not having to travel to the clinic and the reduction of unnecessary in-person visits. The challenges of conducting telemedicine virtual visits were discussed in 45% of the interviews/focus groups. Major concerns were related to technical glitches with audiovisual connections; determining which types of chief complaints were appropriate for virtual vs in-person visits; patient focus, engagement, and safety during visits if conducted outside of the home (eg, in their car, at work, other places); and the need for a care team workflow redesign to reduce patient wait times for virtual visits.

**Table 4.** Patient and Provider Perceptions of Telemedicine

Thematic Domain	Patient^a^	Primary Care Provider
Attitude	*User*: I like the virtual because you don’t have to leave your home. You don’t have to get dressed.	You don’t need to drive the 20 minutes to the clinic and wait for an hour in the waiting room for me to spend three minutes in the room and say, “Yeah, your toenail is infected. I’m going to send some antibiotics and do some warm soaks. Bye.” … that's like three hours out of their day for me to tell them that.
Behavioral intention	*User*: But if it's going over results or something, or just going over something, then the video is fine. But, like, if I’m seeing them for the first time or they’re doing, obviously, some kind of test, or I have a concern, I’d rather be face-to-face.	I’ve got some repeat business on telemedicine, so some of them really like it. Some of them, I offer it and they say, “No, I’d rather come in person.” So we thought that when COVID was kind of dying down and we went back to in-office visits that we would continue to have a large portion of virtual visits and it really hasn’t panned out as much as we thought it would.
Perceived behavioral control (subdomain facilitating conditions)	*User*: I tried that once. But the video would not connect. So I ended up talking to him over the phone.	They all start out as audio-visual but if the connection's bad, sometimes you’ll end up just calling them for the rest of the visit.
Perceived usefulness	*User*: I’m able to see the doctor one-on-one. It's not physically in person, but it is. I’m able to talk to him…if I talk to him on the phone, just talking, sometimes it doesn’t do what it would do if I was talking to them through virtual. You can see when they go to talk to you. It's just better that way, it seems.	I mean, it's definitely a useful tool when it's used for the right reasons…medication follow-ups, established problems…Now, of course, if they have an MSK problem and it's something that requires an exam, I think it's just all knowing when to use it.
Social influence		The one thing that's good that's coming out of the pandemic though is more of my older patients know how to video chat with their family now using their phone. Family members have even sent them phones to video chat. So, they know how to use the phone, how to text, and how to video chat. That's assisted during visits for me to be able to talk to family so that they understand things as well.
Other–wait time/workflow	*User*: But I found that many of them were not punctual with the time that they were supposed to be on the visit. They were always, I don’t know, I found that mine were always a little bit late.	My biggest gripe with telehealth is it's sometimes hard to communicate with the patient, like, that I’m coming, if I’m running a little behind or something. If you can’t just do virtual or in-person, that's almost impossible unless you’re really good about staying on time.

^a^*User* indicates that the patient uses digital technology, defined as patient portal, chronic disease remote monitoring, and/or telemedicine.

COVID, coronavirus disease 2019; MSK, musculoskeletal.

## DISCUSSION

Study participants (patients and PCPs) valued digital health solutions. Patients reported having mobile devices to access the technology, but they face digital literacy and infrastructure barriers to equitable utilization of the technology (eg, access to high-speed broadband). Provider endorsement appears to influence patient engagement with digital health technology. PCPs voiced concerns about increasing workloads and workflow disruptions. Providers’ experience with workload imbalance could temper their enthusiasm for incorporating digital technology in their clinical practice and limit their endorsement of it for patient use.

Technology is becoming increasingly important for accessing health care, self-care tools, and health information. However, a digital divide remains between those with and without access to technology. According to Pew Research Center population survey studies, while the overall rate of internet usage has increased over time, there are age, education, and income-related gaps in who is using the internet.^15,16^ Moreover, there are disparities in access to broadband service at home across racial minorities and individuals with lower levels of income and education. Individuals with less than a high school education, those with lower incomes, and younger adults are more likely to rely on smartphones for online access. US adults in lower income households have lower levels of technology uptake^15,16^; in households with an income <$30,000 vs households with an income >$100,000, technology uptake is as follows: smartphone, 76% vs 97%; desktop or laptop computer, 59% vs 92%; broadband, 57% vs 93%; and tablet computer, 41% vs 68%, respectively. Complicating matters further are sociodemographic differences in digital readiness (ie, confidence in one's ability to use technology).^17^

Within this larger social context, an imperative for health systems is to implement user-centric strategies for facilitating uptake of technology while mitigating the risk of worsening the digital divide. The authors recommend systematically assessing patients’ resources for accessing technology as a social determinant of health. This assessment should include documenting whether patients have a smartphone, and if so, how they use it (eg, phone calls only, surfing the internet, managing bills). Additionally, use of other devices such as computers and tablets should be recorded, as well as patients’ preferred location for accessing the internet or Wi-Fi (eg, home, work, public library, other). Every patient should be asked whether they feel unskilled and/or need help with using digital devices or technology as this information may provide insights into their level of digital literacy. Health systems should also assess patients’ hesitancy or preference for using technology to manage their care. Ideally, the workflow for capturing this information would be integrated into procedures for assessing social determinants of health.

Study findings suggest that health system operational definitions and corresponding measures for level of patient engagement in using digital health interventions are needed. Doing so permits tracking patterns of use across subpopulations to identify opportunities for targeted education and outreach to patients who appear to be less engaged. Ideally, selection of subpopulations to monitor should be data-driven, based on local trends. Nonetheless, factors known to influence patient portal utilization include age, race, ethnicity, degree of comorbidity, education level, health literacy, attitudes/preferences for using technology, and patient preferences for how to access services.^18-20^ These predictors of utilization substantiate the need to systematically collect such information as a standard practice.

Provider education is needed about the power of messaging the positive value of technology for care delivery and self-management. For example, regarding RPM, Walker et al suggest that patients may value increasing their disease-specific knowledge, triggering earlier clinical assessments and treatment, improving self-management, and enhancing shared decision-making.^21^ These benefits of RPM could be incorporated into discussions when referring patients for enrollment. Regarding telemedicine (video teleconferencing), Fischer et al suggest that demographic differences in use may reflect differences in willingness to use it.^22^ Fear of losing interpersonal contact and increased burdens associated with learning something new, increased out-of-pocket costs, and lack of trust in technology may temper perceived benefits. Therefore, acknowledgement of concerns and reassurance about what procedures are in place to address these concerns are equally important for building trust in the value of such programs.

Providers are likely to avoid messaging of any kind if the perceived net result is increased workloads and work inefficiencies. In this study, providers resoundingly emphasized the importance of care team triage for managing electronic medical record system tasks which include responding to patient portal messages. Regarding telemedicine, providers recommended designing schedule templates so that clinic sessions are devoted to only 1 type of visit, in-person clinic or telemedicine only.

In 2022, the American Medical Association published a Digital Health Implementation Playbook Series that provides a tactical approach to planning, executing, and evaluating the success of telehealth programs.^23^ Workflow redesign that captures the entire life cycle of a visit (before, during, and after) is paramount. A good workflow makes the process easier for patients, providers, and staff. Telehealth must also be inclusive: (1) identifying community resources for patients who may have challenges accessing technology, (2) incorporating medical interpreters in the telemedicine visit, (3) providing educational and technical support to patients as needed, and (4) keeping the caregiver as part of the process.

### Limitations

This qualitative study has several limitations that may limit external generalizability of the results. This study was conducted during the COVID-19 pandemic which may have influenced uptake of technology use because of concerns about overall public health safety. Study participants were recruited through a variety of methods (eg, management recommendation, convenience sampling), so patient and provider perceptions captured in the interviews may reflect response bias in favor of or against technology. Nonetheless, our study findings confirm perceptions previously reported in the literature. Most patients and providers successfully recruited were from urban areas with fewer representing rural areas. In the midst of the pandemic with resource limitations, prioritizing this research study was difficult for the rural clinics. The experience of non-English-speaking patients and individuals with limited English proficiency using technology was not captured. Future studies must target these underrepresented populations (eg, rural, limited English proficiency) for further exploration. Finally, patient access to and use of RPM for chronic disease management varied geographically based on when the services became available.

## CONCLUSION

Most study participants had positive views about the use of technology to manage health. Therefore, facilitating conditions such as availability of resources for using technology (proxy user, education on how to set up and/or use, internet/Wi-Fi) is critical. Some features of patient portal functionality are convenient and desirable for patients but may inadvertently increase provider workloads. Remote monitoring technology was uniformly seen as useful, but uptake may be facilitated/hindered by insurance coverage of devices, literacy (health and technology), internet/Wi-Fi access, and aspects of programming that may be engaging or disengaging. Uptake of telemedicine is largely influenced by access to high-speed broadband which affects the quality of video teleconferencing.

## Notes

*This article meets the Accreditation Council for Graduate Medical Education and the American Board of Medical Specialties Maintenance of Certification competencies for Patient Care, Medical Knowledge, and Systems-Based Practice.*
